# Bone Marrow-Derived CD44^+^ Cells Migrate to Tissue-Engineered Constructs via SDF-1/CXCR4-JNK Pathway and Aid Bone Repair

**DOI:** 10.1155/2019/1513526

**Published:** 2019-07-24

**Authors:** Yanzhu Lu, Junchao Xing, Xiaolong Yin, Xiaobo Zhu, Aijun Yang, Jiyue Luo, Jing Gou, Shiwu Dong, Jianzhong Xu, Tianyong Hou

**Affiliations:** ^1^National and Regional United Engineering Laboratory of Tissue Engineering, Department of Orthopedics, Southwest Hospital, Third Military Medical University, Chongqing, China; ^2^Center of Regenerative and Reconstructive Engineering Technology in Chongqing City, Chongqing, China; ^3^Tissue Engineering Laboratory of Chongqing City, Chongqing, China; ^4^Outpatient Department of 31668 Unit of PLA, China; ^5^Department of Biomedical Materials Science, School of Biomedical Engineering, Third Military Medical University, Chongqing, China

## Abstract

**Background and Aims:**

Host-derived cells play crucial roles in the regeneration process of tissue-engineered constructs (TECs) during the treatment of large segmental bone defects (LSBDs). However, their identity, source, and cell recruitment mechanisms remain elusive.

**Methods:**

A complex model was created using mice by combining methods of GFP^+^ bone marrow transplantation (GFP-BMT), parabiosis (GFP^+^-BMT and wild-type mice), and femoral LSBD, followed by implantation of TECs or DBM scaffolds. Postoperatively, the migration of host BM cells was detected by animal imaging and immunofluorescent staining. Bone repair was evaluated by micro-CT. Signaling pathway repressors including AMD3100 and SP600125 associated with the migration of BM CD44^+^ cells were further investigated. *In vitro*, transwell migration and western-blotting assays were performed to verify the related signaling pathway. *In vivo*, the importance of the SDF-1/CXCR4-JNK pathway was validated by ELISA, fluorescence-activated cell sorting (FACS), immunofluorescent staining, and RT-PCR.

**Results:**

First, we found that host cells recruited to facilitate TEC-mediated bone repair were derived from bone marrow and most of them express CD44, indicating the significance of CD44 in the migration of bone marrow cells towards donor MSCs. Then, the predominant roles of SDF-1/CXCR4 and downstream JNK in the migration of BM CD44^+^ cells towards TECs were demonstrated.

**Conclusion:**

Together, we demonstrated that during bone repair promoted by TECs, BM-derived CD44^+^ cells were essential and their migration towards TECs could be regulated by the SDF-1/CXCR4-JNK signaling pathway.

## 1. Introduction

Large segmental bone defects (LSBDs) resulting from severe trauma, tumor resection, and debridement after infection are common and challenging in clinical settings [1, 2]. At present, the effectiveness of tissue-engineered constructs (TECs), which are constituted by incorporating viable osteogenic progenitors (most usually mesenchymal stem cells (MSCs)) into 3D biocompatible scaffolds and then delivering them to LSBDs, has been proved by mounting evidence, including clinical trials with high confidence levels [3, 4]. Considering the unique pluripotency and regenerative properties of MSCs, early studies deservedly suggested that seed cells differentiated into osteogenic cells and facilitated bone repair in a direct way. However, recent studies using stem-cell-tracing techniques have confirmed that most of the seed cells die or disappear *in vivo* for a short time. Only a part of them survive and participate in the eventual osteogenesis [5]. To date, the indirect paracrine effect, by which donor MSCs modulate the local microenvironment at implantation sites to attract host cells, but not direct differentiation or supplementary roles, has been accepted as a major functional mechanism [6–8]. However, the identity, source, and locomotor mechanism of the host cells involved remain elusive.

After tissue injury, stem cells are mobilized out from bone marrow (BM) and recruited to the injury area via peripheral circulation. A similar characteristic has been exhibited by MSCs [9]. Although the proportion of MSCs in BM accounts for only 0.001% to 0.01% of monocytes and equivalent to 1/10 to hematopoietic stem cells (HSCs), MSCs proliferate abundantly and rapidly migrate to injury sites in response to injury signals [10]. Based on this specialty and the multilineage differentiation capacity, they have been intensively studied and applied as powerful therapeutic tools for a variety of diseases and conditions, including LSBDs [11, 12]. Automatically, it is easy to speculate that MSCs may be an integral part of host cell populations involving TEC-mediated bone repair. However, MSCs constitutively express various surface markers, such as CD73, CD90, and CD105, and lack CD45, CD34, CD14 or CD11b, CD79*α* or CD19, and HLA-DR surface molecules, making the tracing extremely difficult, especially *in vivo* [13]. It is not known yet if MSCs with a positive marker can migrate to a bone defect. Among the adhesion molecules, the CD44 marker, a widely expressed cell surface hyaluronan receptor, is famous for interacting with matrix metalloproteinases (MMPs) to regulate cell-cell and cell-matrix interactions and mediate cell mobilization [14]. Thus, tracing host cells expressing CD44 may shed important insights into the molecular mechanisms underlying TEC-induced bone repair. As known, BM is the largest stem cell bank and multiple osteoprogenitors reside therein, making BM highly suspected to serve as an inestimable source of host cells which are involved in TEC-induced bone repair.

For these reasons, we hypothesized that host cells involved in eventual TEC-induced bone repair would probably originate from BM and CD44^+^ populations and may play important roles therein. Up to now, an effective animal model to reach this goal is absent. In view of this, a compound model of GFP^+^ bone marrow transplantation (GFP-BMT), mouse parabiosis, and femoral LSBD was designed. The parabiosis which establishes common blood circulation between 2 surgically-joined mice has provided an excellent model for investigating various biological processes including the involvement of nonresident stem and hematopoietic cells migrating to the injury site in tissue repair and regeneration. During BMT, the hematopoietic system of the recipient mouse is destroyed by lethal irradiation. The following transplantation of exogenous GFP^+^ BM cells allows the trace of BM-derived cells. With the help of the combined model, we preliminarily explored the host cell motion events after implantation of TECs.

## 2. Materials and Methods

### 2.1. Animals

In total, 38 wild-type (w/t) FVB/N mice and thirty-three 8-week-old FVB/N transgenic green fluorescent protein (GFP^+^) mice were purchased from the animal center of the Third Military Medical University, Chongqing, China. All animal experiments were approved by the Institutional Animal Care and Use Committee of the Third Military Medical University.

### 2.2. Cell Isolation and Expansion

Mouse bone marrow mesenchymal stem cells (mBMSCs) were obtained from FVB/N mice as described previously [15]. Briefly, cells from flushing solutions of the femora and tibia were cultured in *α*-Dulbecco's modified Eagle's medium (*α*-DMEM, *α*-DMEM with 10% fetal bovine serum, HyClone Laboratories Inc., South Logan, UT, USA) supplemented with 100 U/ml penicillin/streptomycin (basic culture medium, BCM). Cells were routinely passaged while reaching 80-90% confluency. Only cells of passage 4 were used in this study. The cells homogeneously expressed markers of MSCs and could differentiate into osteoblasts and lipoblasts, indicating their MSC characteristics [16].

### 2.3. Fabrication of TECs

Decalcified bone matrix (DBM) scaffolds were chosen as cell carriers due to their excellent capacities of supporting the adhesion, growth, and proliferation of MSCs [16]. They were prepared using porcine trabecular bones from Yunnan miniature pigs according to a previously described method [17]. TECs were fabricated by dropwise instilling an aliquot (20 *μ*l, 1 × 10^6^ cells/ml) of a single mBMSC suspension onto the two opposite surfaces of DBM. After 2 hours of standing for cell penetration, culture media were added and then changed every 3 days. TECs harvested on day 10 were used for implantation.

### 2.4. BMT

**Figure 1:**
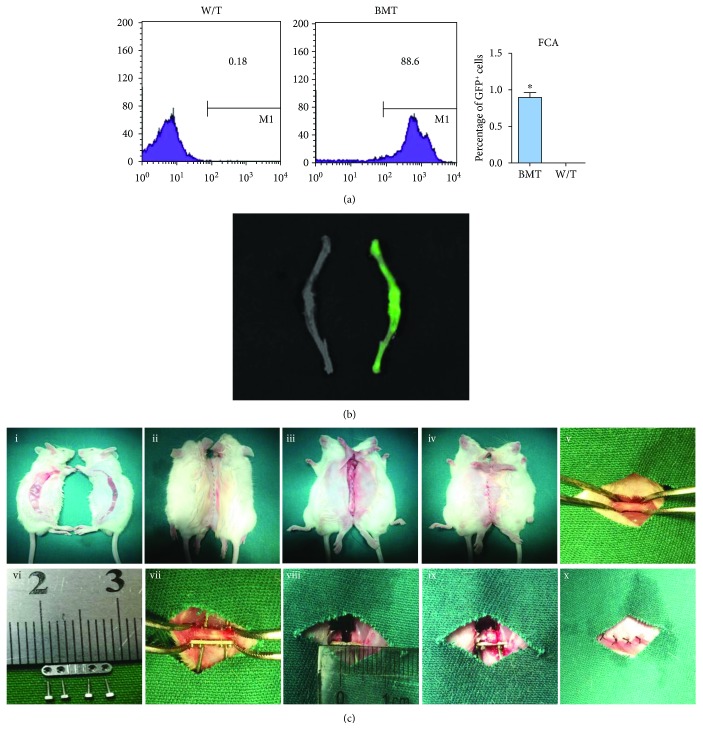
Bone marrow transplantation (BMT) and the surgical procedures of complex animal models. (a) At 6 weeks, GFP^+^ cells in bone marrow of BMT and wild-type mice were detected by flow cytometry. The numerical value represents the mean percentage of GFP^+^ cells in each group (*n* = 3). (b) Bioluminescence image of femur and tibia. Green fluorescence intensity was observed between wild-type (left) and BMT (right). (c) The parabiotic mouse model was fabricated (i-iv) and two weeks later, a critical-sized bone defect was created (v-x).

Under aseptic conditions, BM cells were isolated from male GFP^+^ transgenic mice. Female w/t mice were lethally irradiated with 8 Gy. After 6 hours, recipient mice received an injection of BM cells (5 × 10^6^ GFP^+^ BM cells/mouse). To check the result, the proportions of GFP^+^ cells in BM were detected in w/t and BMT mice at 6 weeks. Three mice were randomly chosen and subjected to flow cytometry analysis (FCA) and the CRI Maestro 500FL In Vivo Imaging System (IVIS). As expected, the proportions of GFP^+^ cells in BM of w/t mice were all less than 1%, while in BMT mice, they were as high as about 88.6% (Figure 1(a)). A similar difference was revealed by IVIS (Figure 1(b)). Therefore, all BMT mice were used at least 6 weeks after BMT.

### 2.5. Parabiotic Mouse Model

Because of sharing all major histocompatibility antigens, parabiotic pairs are free of immunological barriers to cell migration [18]. To establish the parabiotic model, pairs of same-age and weight-matched female BMT and w/t mice were raised together for 2 to 3 weeks. Parabiotic partners were then joined surgically by a modification of the Bunster and Meyer technique (Figure 1(c)). Briefly, matching skin incisions were made on each mouse from the olecranon to the knee-joint and about 0.5 cm free skin was exposed by blunt separation of the subcutaneous fascia (Figure 1(c)-i). The elbow and knee joints were then fixed, respectively. The dorsal and ventral skin incisions were successively sutured with connective tissues of the two mice approximately (Figure 1(c)-ii–iv). Postoperative mortality was nil, and the health of animals after parabiosis was excellent.

Two weeks after parabiotic joining, a 2 cm defect was developed in the middle femoral shaft of each w/t mouse as previously described by us [19]. After sufficient exposure of soft tissues, the periosteum was carefully erased from the trochanter to the condyle, with the distal articular capsule preserved (Figure 1(c)-v). A segmental defect centered on the femoral shaft was then created using a self-designed plate screw system (Figure 1(c)-vi). Using a 0.7 mm aiguille, four holes penetrating the entire femur were drilled along the holes. The plate was tightly fixed to the femur by four steel screws (Figure 1(c)-vii). Then, approximately 2 mm of the femoral shaft was removed using a dental grinding drill (Figure 1(c)-viii). Thereafter, TECs or DBM scaffolds were trimmed to appropriate sizes and implanted into the defects via press fitting (Figure 1(c)-ix). The implants were further fixed by decussate sutures. The muscle and skin were closed layer by layer (Figure 1(c)-x). Postoperatively, the general health and activity were monitored daily for 2 weeks.

### 2.6. In Vivo Migration Assay

On days 1 and 3 postoperatively, 3 mice from each group were sacrificed and femora were collected. After fixation in 4% paraformaldehyde, samples were immediately subjected to the Xenogen IVIS Spectrum Imaging System (PerkinElmer, Waltham, MA, USA). Subsequently, immunofluorescent staining was performed. After decalcification with EDTA, frozen sections (6 *μ*m thick) were prepared with a cryostat (Leica Microsystems AG, Wetzlar, Germany), permeabilized with 0.3% Triton X-100, and blocked with normal donkey serum (1 : 20; Huayueyang Biotechnology, Beijing, China). Then, slides were incubated with primary rabbit anti-CD44 (1 : 250; Abcam, Cambridge, UK) overnight at 4°C and stained with the secondary donkey anti-rabbit-Cy3 (1 : 50; Jackson ImmunoResearch Inc., USA) for 1 hour. All sections were counterstained with 4′,6-diamidino-2-phenyilindole (DAPI; Invitrogen, USA) for 10 min. Relative cellularity was determined for each harvested implant using a confocal laser scan microscope (CLSM; Leica Biosystems, Wetzlar, Germany). GFP^+^ host cells were further identified by DAPI-labeled nuclei and for each section, the number of GFP^+^/CD44^+^ cells was counted in 3 random high-power fields (hpfs).

Besides, peripheral blood (PB) and BM were obtained from the tail and long bones, respectively. The concentrations of SDF-1 were measured via ELISA and compared. After erythrocyte lysis and fixation, CD44^+^ cells were sorted from PB and analyzed by fluorescence-activated cell sorting (FACS).

In addition, to evaluate the roles of signaling molecules, AMD3100 (5 mg/kg; Sigma-Aldrich, USA) or SP600125 (30 mg/kg; Sigma-Aldrich, USA) was intraperitoneally injected into mice receiving TEC implantation once a day postoperatively. On day 3, PB and TECs were harvested and subjected to FACS and immunofluorescent staining, respectively.

### 2.7. RT-PCR

CD44^+^ cells sorted from PB were subjected to RNA analysis. Total RNA was extracted using the TRIzol Reagent (Invitrogen, Carlsbad, CA), and cDNA was prepared from 10 *μ*g of total RNA by using reverse transcriptase with an oligo-dT primer according to the manufacturer's instructions (Promega Corporation, Madison, WI, USA). All reactions were carried out using the SYBR Green Mix (Takara Bio Inc., Nojihigashi, Japan). qRT-PCR was carried out using the CFX96 Touch q-PCR System (Bio-Rad, Hercules, CA). All reactions were run in triplicate and were normalized to the housekeeping gene GAPDH. The primers used are listed in Table 1.

### 2.8. Bone Repair Assay

At 4 weeks postoperatively, 6 pairs of parabiotic mice were euthanised and the femurs of w/t partners were excised and the internal fixation device was carefully removed. Then, samples were analyzed using a micro-CT (Viva CT40, Scanco Medical AG, Bassersdorf, Switzerland). The cross section of the femoral graft area was subjected to analyze osteogenic indices.

### 2.9. In Vitro Migration Assay

Confluent mBMSCs (equivalent to donor MSCs) were harvested and incubated with BCM supplemented with 4 ng/ml IL-1*β*, 10 ng/ml IL-6, and 20 ng/ml TNF-*α* (all from PeproTech Asia, USA), which were then used to simulate an inflammatory microenvironment *in vivo*. After 48 hours, the supernatants were collected, centrifuged, and aliquoted to prepare conditioned media of MSCs (MSC-CM).

Migration assays were performed in transwell systems (8 *μ*m pores; Corning Costar Corp., USA). Different inducing media (700 *μ*l; see Tables 2 and 3) were added into the bottom compartment. CD44^+^ BM cells were obtained by sorting BM cells with CD44-PE antibody (eBioscience, San Diego, CA, USA) using FACS. They were preincubated with BCM or BCM supplemented with different inhibitors (Tables 2 and 3). Then, they were loaded into the upper chambers at approximately 1 × 10^5^ cells/chamber and allowed to migrate towards distinct inducers for 15 hours. Then, cells on the upper side (nonmigrating cells) were removed and migrated cells on the lower face were washed with PBS, fixed with 4% paraformaldehyde (Boster Biological Technology, Wuhan, China), and stained with DAPI. Under a microscope, the number of migrated cells was counted on 5 random high-power fields (200x magnification) and averaged. The migration assay was repeated 3 times for each cell batch. At the same time, cells retained in transwell chambers were collected for further protein analysis.

### 2.10. Western Blot Analysis

CD44^+^ BM cells harvested from transwell systems were lysed with SDS lysis buffer (100 mM Tris at pH 8.0, 10% glycerol, and 1% SDS) and protein concentration was determined using a NanoVue Spectrophotometer (GE Healthcare Life Sciences, USA). A total of 30 *μ*g of protein lysates was separated by SDS-PAGE (80 V, 120 min; Beyotime, Shanghai, China) and transferred to polyvinylidene difluoride membranes (250 mA × 60 min, Millipore, USA). After blocking with 5% milk, membranes were incubated overnight at 4°C with the following primary antibodies: anti-CXCR4, anti-p-ERK, anti-p38, anti-p-JNK, and anti-Arpin (1 : 1000 dilution; Abcam, USA), and anti-Arp2/3 (1 : 1000 dilution; CST, USA), followed by incubation with horseradish peroxidase-conjugated secondary antibody (1 : 2000 dilution; SouthernBiotech, Birmingham, AL) at room temperature for 1 hour. Signals were detected by enhanced chemiluminescence (KPL, Gaithersburg, MD). GAPDH was used as the loading control. Western blot was repeated 3 times for each cell batch.

### 2.11. Statistical Analysis

Data are expressed as mean ± standard deviation (SD). The significant differences were analyzed by one-way ANOVA followed by an LSD *t*-test. The statistically significant level of differences was set at *P* < 0.05.

## 3. Results

### 3.1. Construction of the Combined Animal Model

**Figure 2:**
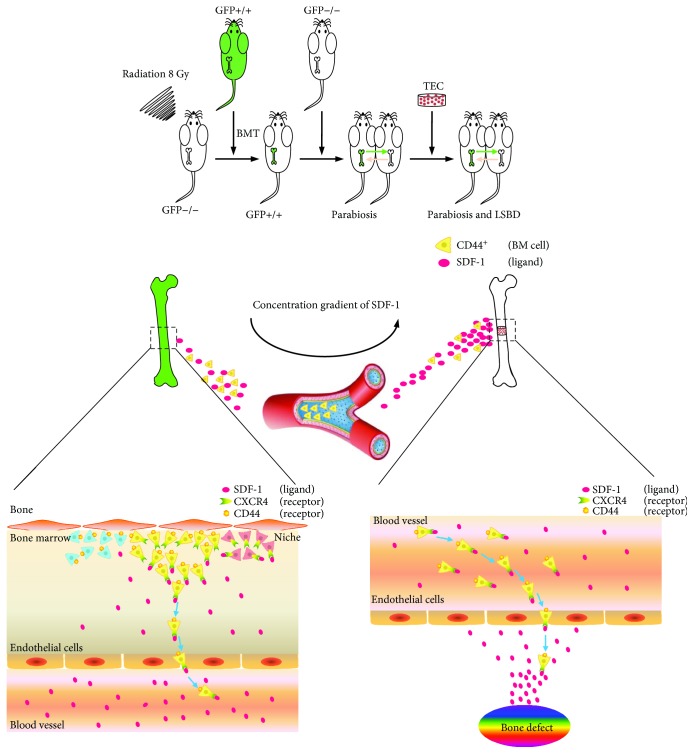
The schematic diagram of the whole research.

In each mouse, BMT lasted 20 min and the mortality rate was 3.7%. The operative procedures, including parabiosis and LSBD, from anesthesia induction to skin closure, lasted 15 min and 20 min, respectively. All mice were awakened within 30 min postoperatively. Beyond lighting, no other postoperative care was required. Postoperative daily monitoring for 3 days showed satisfactory health conditions and no change in activity, temperament, or vocalization in any case. Although weight bearing was reduced for several days after LSBD modeling, the gait pattern returned to normal in each parabiotic pair after 10 days. Postoperative recovery was uneventful, except that two mice died of unknown reasons on day 17 and 22 which were then immediately supplemented. The mortality rate of surgeries was 4.3%. These findings indicated the reproducibility and availability of the combined animal model. The schematic diagram of the whole research is provided in Figure 2.

### 3.2. BM-Derived CD44^+^ Cells Migrated to TECs to Promote Bone Repair

**Figure 3:**
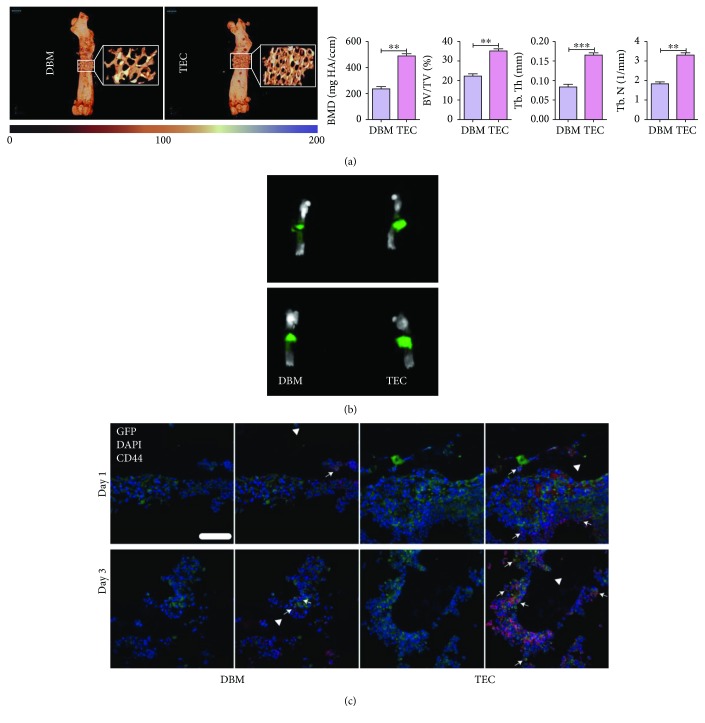
Imaging examination and *in vivo* recruitment of BM cells towards different implants. (a) A more calcified bone matrix was observed in the TEC group compared to that in the DBM group by micro-CT. The osteogenic parameters of TECs were all significantly better than DBM. (b) Representative images from IVIS. The implantation sites of TECs showed higher fluorescence intensity than DBM (days 1 and 3). (c) Representative images of *in vivo* migration of GFP^+^/CD44^+^ cells revealed by immunofluorescence staining. Postoperatively, more GFP^+^ and GFP^+^/CD44^+^ cells emerged in TECs than in DBM. TECs, tissue-engineered constructs; white triangle, implant area; white arrows, GFP^+^/CD44^+^ cells; scale bar, 50 mm. ^∗∗^*P* < 0.01.

Firstly, we echoed the previous finding that compared with DBM scaffolds, MSC-incorporated TECs owned superiority in bone repair (Figure 3(a)). IVIS showed that on postoperative day 1, more intense GFP fluorescence accumulated in the region of the TECs, as compared with DBM. The fluorescence intensity increased with significant distinction on day 3 (Figure 3(b)). Consistently, immunofluorescent staining demonstrated that more GFP^+^ cells emerged at the TEC site on day 1. As time extended, more GFP^+^ cells were recruited at implantation sites but the difference between TEC and DBM was still remarkable. Interestingly enough, most of the GFP^+^ cells in implants were CD44 positive and the number of GFP^+^/CD44^+^ cells was substantially larger in TECs than in DBM (Figure 3(c)). In combination with the osteogenic difference, the majority of host cells recruited to promote TEC-mediated bone repair were CD44-positive BM cells.

### 3.3. The SDF-1/CXCR4-JNK Pathway Mediated the Migration of GFP^+^/CD44^+^ BM Cells

**Figure 4:**
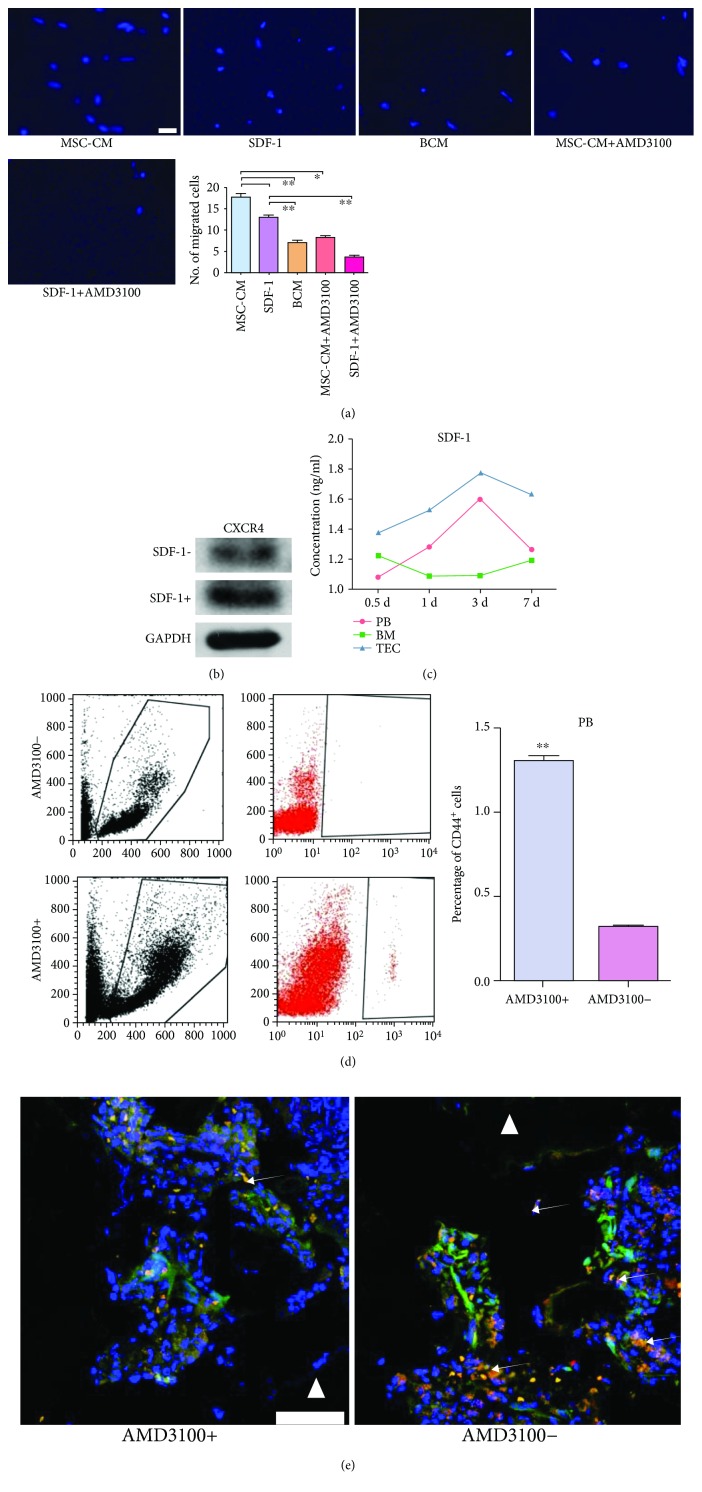
The SDF-1/CXCR4 axis promoted the migration of GFP^+^/CD44^+^ bone marrow (BM) cells. (a) Representative images of migrated BM cells in different groups. The quantification of the migrated BM cells is shown as a bar graph (*n* = 5). ^∗^*P* < 0.05. ^∗∗^*P* < 0.01. (b) Comparison of CXCR4 protein expression after SDF-1 induction. After migration stopped, BM cells were collected and analyzed by western blot. (c) Postoperatively, the concentration of SDF-1 in different tissues was measured daily using ELISA. The different tendency is presented on the line chart. (d) FACS analysis of the proportions of CD44^+^ cells in PB. Postoperatively, cells collected from peripheral blood (PB) of mice receiving AMD3100 or not were incubated with fluorescently conjugated antibodies against CD44 and analyzed using the CytoFLEX software. The quantification comparison is shown as a bar graph (*n* = 3). ^∗∗^*P* < 0.01. (e) Representative images of *in vivo* migration of GFP^+^/CD44^+^ cells towards TECs. The recruitment of GFP^+^/CD44^+^ cells was significantly reduced after systematic delivery of the AMD3100 group. White triangle, implant area; white arrows, GFP^+^/CD44^+^ cells; scale bar, 50 mm. TECs, tissue-engineered constructs. MSC-CM, conditioned media of mesenchymal stem cells. BCM, basic culture medium.

Stromal cell-derived factor-1 (SDF-1) and its receptor were widely accepted as crucial regulators in the mobilizing and trafficking of multiple stem cells, such as HSCs, MSCs, and endothelial progenitor cells (EPCs) [20]. Considering that stem cells might occupy a substantial proportion in the recruited host CD44^+^ BM cells, the influence of SDF-1 and its receptor CXCR4 were evaluated. *In vitro*, SDF-1 exhibited analogous chemotactic power to MSC-CM. Chemotactic effects of MSC-CM and SDF-1 were abolished by AMD3100, an antagonist for CXCR4 (Figure 4(a)). Meanwhile, the protein expression of CXCR4 in migrated cells was remarkably enhanced by SDF-1 (Figure 4(b)). *In vivo*, the levels of SDF-1 in PB and TECs gradually increased and peaked on day 3, showing a similar tendency to the recruitment of host CD44^+^ BM cells. The SDF-1 level in BM showed an almost inverse variation tendency (Figure 4(c)). These collectively suggested that the SDF-1/CXCR4 axis elicited crucial effects on the movement of host CD44^+^ BM cells. However, the systematic introduction of AMD3100 led to opposite chemotactic effects between PB and TECs. The number of GFP^+^/CD44^+^ cells in PB was significantly elevated in response to AMD3100 but was decreased in TECs (Figures 4(d) and 4(e)). This might be attributed to the unique promoting effect of AMD3100 on BM mobilization. Overall, the SDF-1/CXCR4 pathway mediated the trafficking of host CD44^+^ BM cells.

**Figure 5:**
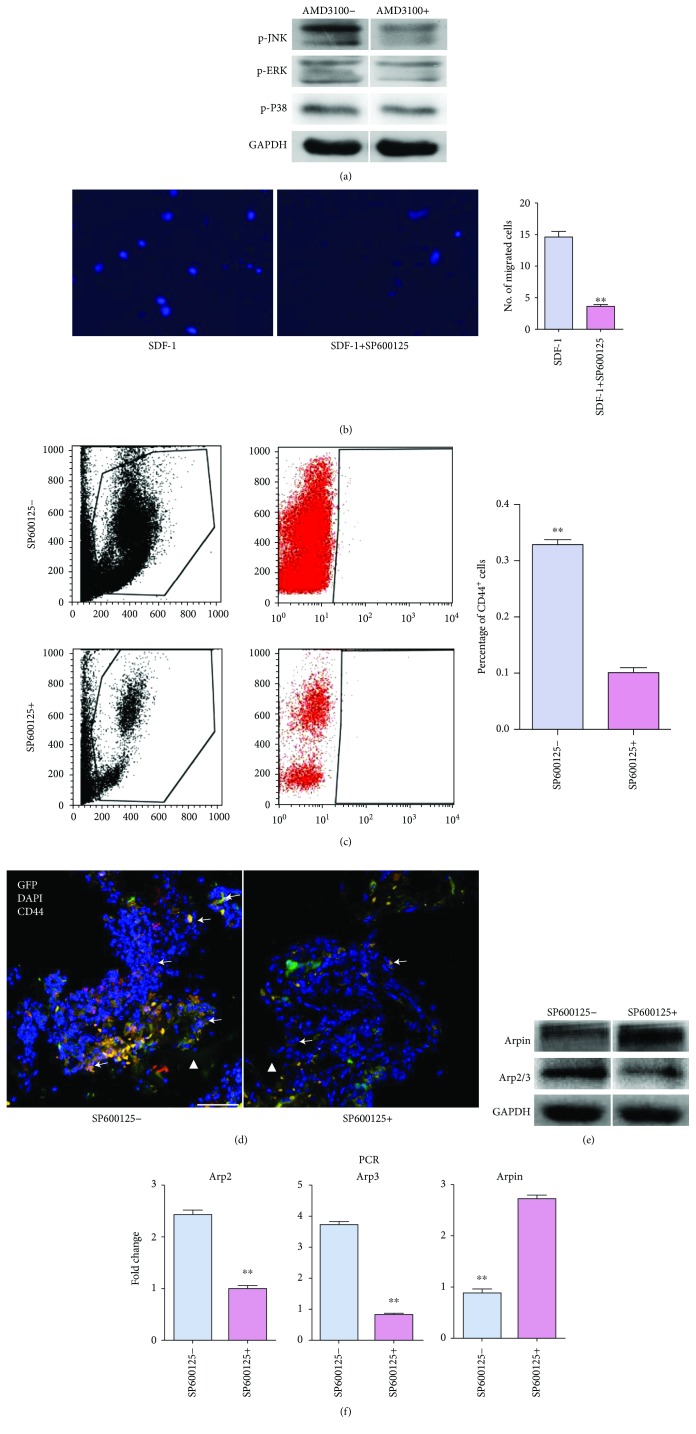
JNK served as an effector downstream of SDF-1/CXCR4 in the migration of bone marrow (BM) CD44^+^ cells towards tissue-engineered constructs (TECs). (a) Comparison of phosphorylated JNK (p-JNK), ERK (p-ERK), and P38 (p-P38) expression in BM cells after CXCR4 blockade. After migration stopped, BM cells were collected and analyzed by western blot. (b) Representative images of cell migration towards SDF-1. The quantification of migrated BM cells is shown as a bar graph (*n* = 5). ^∗∗^*P* < 0.01. (c) FACS analysis of the proportions of CD44^+^ cells in peripheral blood (PB). The quantification comparison is shown as a bar graph (*n* = 3). ^∗∗^*P* < 0.01. (d) Representative images of *in vivo* migration of GFP^+^/CD44^+^ cells towards TECs. The introduction of SP600125 significantly reduced the recruitment of GFP^+^/CD44^+^ cells. White triangle, implant area; white arrows, CD44^+^ cells; scale bar, 50 mm. (e) Comparison of Arpin and Arp2/3 expressions in BM cells after JNK blockade *in vitro*. (f) Analysis of the Arpin, Arp2, and Arp3 mRNA expression in sorted CD44^+^ cells. On day 3 postoperatively, cells in PB were sorted by FACS on CD44. RT-PCR was performed to evaluate Arpin, Arp2, and Arp3 mRNA expression. The quantification data are shown as a bar graph (*n* = 3). ^∗∗^*P* < 0.01.

To identify the effectors downstream of SDF-1/CXCR4, the MAPK signaling pathway was chosen as a candidate due to its possible association with SDF-1 and stem cell migration [21]. *In vitro*, SDF-1 activated JNK, ERK, and P38. However, only the expression of p-JNK in migrated cells were significantly reduced by AMD3100 (Figure 5(a)). Moreover, the introduction of SP600125, a highly selective JNK-antagonist, substantially inhibited the chemotactic effect of SDF-1 (Figure 5(b)). This indicated that JNK served as an effector downstream of SDF-1/CXCR4. *In vivo*, the numbers of both PB-circulating and TEC-recruited CD44^+^ BM cells were substantially reduced by SP600125 (Figures 5(c) and 5(d)). Meanwhile, the protein expressions of Arpin and Arp2/3 in migrated cells were up- and downregulated by SP600125, *in vitro* (Figure 5(e)). Consistently, the gene level of Arp2/3 was significantly lowered and Arpin was elevated in CD44^+^ BM cells by SP600125 (Figure 5(f)). Collectively, the aforementioned findings illustrated that the migration of host CD44^+^ BM cells to TECs were mediated by the SDF-1/CXCR4-JNK pathway.

## 4. Discussion

The advantages of MSC-based TECs over blank scaffolds in repairing LSBDs have been proven both basically and clinically, highlighting the important roles of implanted donor MSCs [12, 22]. Previously, researchers ascribe the superiority of TECs to the direct replenishment of osteoprogenitors. However, emerging literature suggests that most of the seeded MSCs are not involved in the final osteogenesis [3, 5]. Tasso et al. have even found that the newly-formed bones in TECs are entirely of host origin [23]. These findings lead to more attention to the paracrine actions of MSCs; that is, secreting a variety of chemokines to attract host cells associated with tissue regeneration [8, 24]. In this context, the concept of in situ tissue engineering strategy has sprung up and remarkable progress has been made [25]. To date, unlike donor MSCs, little is known about the host cells involved in TEC-mediated bone repair. Better understanding of this issue will shed light on the development and application of MSC-based strategies.

To figure out the origin of host cells, a suitable animal model should be created. Initiatively, we combined BMT, parabiosis, and LSBD modeling approaches in one mouse model. In this model, lethal irradiation destroyed the hematopoietic system of the mouse and the transplantation of exogenous GFP^+^ BM cells was employed to complete a significant replacement in BM [26]. Another wild-type mouse was surgically connected with this GFP-BMT mouse to achieve parabiosis. After about 2 weeks, shared circulation was established free of immunological barriers and was allowed between parabiotic partners. Also, cells and soluble factors were interchangeable [27]. Thus, the wild-type mouse can receive BM-derived GFP^+^ circulating cells from the GFP-BMT mouse, making it easy to draw the migration path of BM-derived cells. It is worth noting that this is not confined to BM cells; previous studies have successfully fabricated parabiosis using GFP^+^ and wild-type mice to track the migration of multiple cells, such as dermal fibroblast/myofibroblast progenitors, lung progenitors, and non-BM progenitor cells [28–31]. However, GFP was systemically expressed by cells from GFP^+^ mice, making it difficult to determine whether host cells migrated to the target area derived from the circulatory system or other parts. In this study, BMT mice underwent a process of bone marrow destruction and hematopoietic system reconstruction. The transplantation of GFP^+^ BM cells allowed us to verify the potential BM origin of cells which migrate into the circulation and contribute to bone repair. Thus, both GFP-BMT and parabiosis are indispensable. As with bone defect modeling, we previously established a reliable and reproducible load-bearing, critical-size femoral defect model in mice with the help of a self-designed screw-plate fixation system. This model was successfully applied in basic research in the field of bone tissue engineering [16, 17]. In summary, the main research purpose, that is, to identify the origin of host cells involved in TEC-mediated bone repair, cannot be achieved in the absence of either model of GFP-BMT, parabiosis, or LSBD. Therefore, we utilized a combined mouse model in this study.

Using this model, we found that upon injury, more BM cells were mobilized and recruited to TECs. Accordingly, osteogenic activity in TECs was significantly facilitated. Moreover, the majority of cells in TECs were GFP positive, indicating that host cells accounting for osteogenesis were majorly derived from BM. This finding is consistent with current literature showing that osteoprogenitors, such as MSCs, reside in bone marrow niches and only a fraction of them are mobilized into peripheral blood. The mobilization and homing of MSCs keep a dynamic balance in bone marrow [32, 33]. Upon injury, the balance is struck and MSCs are mobilized into peripheral blood and attracted to the injury site under the guidance of multiple chemokines [32]. Interestingly, a considerable proportion of host cells in TECs expressed CD44, one of the positive markers of MSCs [34]. Being involved in cell-cell and cell-substrate interactions, CD44 class I transmembrane glycoproteins play key regulatory roles in regenerative processes. Indeed, CD44 has been implicated in the regulation of cell survival, proliferation, and migration of MSCs [35, 36]. In response to chemotaxis signals, CD44 is activated by hyaluronan and enhances the level and relocation of MMP-9 on the cell surface, mediating collagen degradation and promoting cell migration. In the present study, most of the recruited BM cells were CD44 positive, indicating that CD44 accounted for the increased recruitment of host BM cells to TECs. Also, this is consistent with previous findings demonstrating that CD44 is responsible for the localization of exogenous MSCs to the injured tissues, such as the kidney, liver, and endothelium [37]. The difference between TEC and DBM groups may be attributed to the regulatory effects of donor MSCs on the local microenvironment. Osteogenesis is an extremely complex and orchestrated process involving a variety of cells, active molecules, and signaling pathways. MSCs occupy a predominant position due to their osteogenic differentiation ability, and their migration to the injury site is an initial indicator of bone regeneration [38]. Although CD44 is constituently expressed by other cell types and regulates the migration of other BM cells, such as T lymphocytes, CD34^+^ stem cells, and HSCs [39–41], our findings support the evidence that osteoprogenitors involved in TEC-induced osteogenesis, mostly MSCs, are mainly derived from BM and CD44 populations and play key roles therein.

One serious problem to the effective implementation of stem cell-based therapy in clinical settings is the low homing efficiency of these cells. Unveiling the mechanism underlying the migration of BM cells will provide more information on the therapeutic improvement. The chemokine SDF-1, also known as CXCL12, has been well documented to affect organogenesis, hematopoiesis, and immune responses via binding to CXCR4. Mounting evidence suggests that the interaction between SDF-1 and CXCR4 mediates the trafficking of multiple myeloma cells *in vivo* [10, 42]. Besides, the SDF-1/CXCR4 axis plays critical roles in both engraftment of HSCs into BM and the mobilization of stem cells from BM niches into PB, where these cells can be delivered to an organ-specific injury site [43]. SDF-1 is produced by multiple stromal and other cells, such as MSCs, osteoblasts, and bone marrow endothelial cells [44–46]. Anyway, the implantation of donor MSCs altered the levels of SDF-1 in BM, PB, and implants to form a concentration gradient. Moreover, the blockade of CXCR4 led to the attenuation in the migration of BM CD44^+^ cells. Concomitant with the diversification of cell types in BM, it is now difficult to identify the major components in BM CD44^+^ cells and in-depth research is required to clarify the relationship between SDF-1/CXCR4 and CD44.

Additionally, we explored whether the MAPK signaling pathways are involved in the migration of BM CD44^+^ cells affected by TECs. The MAPK pathway has been shown to regulate a variety of cellular behaviors including survival, proliferation, and migration [47–49]. There are three major components of MAPK pathways: ERK1/2, P38 MAPK, and JNK. We detected them by western blot and found that they were all activated. No significant difference was observed in p-ERK and p-P38 after treatment with AMD3100. However, the phosphorylation of JNK was markedly inhibited by AMD3100. This is consistent with a previous study reporting that the migration of MSCs can be regulated via the JNK signal pathway [50]. To further illuminate the roles of JNK, the specific inhibitor SP600125 was adopted. During cell migration, protrusion of the plasma membrane is essential to form lamellipodia. Lamellipodial protrusion is powered by actin polymerization, which is mediated by the Arp2/3-induced nucleation of branched actin networks. Recently, advances have been made in our understanding of Arp2/3 regulators in lamellipodium dynamics and cell migration [51]. Arpin, a recently-described negative regulator, binds to the Arp2/3 complex and suppresses actin filament nucleation. As a result, cell migration is inhibited, which is manifested in the decreases of both cell speed and directional persistence [52]. Thus, the expressions of Arp2/3 and Arpin were chosen as potential effectors downstream of JNK in the context of cell migration. Eventually, we found that blockade of JNK resulted in the impairment of BM cell migration, accompanied by a reduction in Arp2/3 expression and elevation in Arpin. Consistent results were obtained from further *in vivo* experiments. Taken together, we concluded that the migration of BM CD44^+^ cells towards TECs was mediated by the SDF-1/CXCR4 signaling pathway in a JNK-dependent manner.

## 5. Conclusion

In conclusion, we combined the GFP-BMT, parabiosis, and LSBD models in mice and demonstrated that donor MSCs incorporated within TECs attracted host BM-derived CD44^+^ cells, which was pivotal to the excellent osteogenic capacity. Moreover, the migration of host BM CD44^+^ cells towards TECs was dependent on the function of SDF-1/CXCR4-JNK axis. Further studies are warranted to expand and specify our insight regarding the donor-host cell interactions and figure out the relationship between SDF-1/CXCR4-JNK and CD44. Based on the findings, it may be possible to develop novel strategies to improve the reparative capacity and cost-effectiveness of TECs and avoid adverse effects.

## Figures and Tables

**Table 1:** Primers used for RT-PCR.

Gene	Species	GenBank ID	Sequence
Arp2	Mouse	NM_146243.2	F: CACATCTTCCCAGCTTTGGT
			R: CAGCTCACTTGCCTCATCAC

Arp3	Mouse	NM_023735.2	F: CAGGCTGTTCTTGCCTTAGC
			R: ATCCTTCAGCCACAGGAATG

Arpin	Mouse	NM_025340	F: GCTTCCGGCTAGGACTGTTAG
			R: CTCGTGTTGGTTAGGCCCAC

GAPDH	Mouse	NM_008085	F: TGGATTTGGACGCATTGGTC
			R: TTTGCACTGGTACGTGTTGAT

**Table 2:** Set-ups in transwell chambers.

Upper	CD44^+^ BM cells

Lower	MSC-CM
	BCM
	BCM + SDF-1
	MSC-CM + AMD3100
	BCM + SDF-1 + AMD3100
	BCM + SDF-1 + SP600125

BM, bone marrow. MSC-CM, conditioned media of mesenchymal stem cells. BCM, basic culture medium.

**Table 3:** Reagents used for *in vitro* experiments.

Reagent	Function	Application	Concentration	Duration	Source
CD44-PE Ab	Cell sorting	Flow cytometry	0.125 *μ*g/test	30 min	eBioscience, USA
CD44 Ab	Cell tracing	Immunofluorescence	1 : 250	12 h	Abcam, Cambridge, UK
SDF-1	Proinflammatory cytokine	Transwell	100 ng/ml	24 h	PeproTech, USA
AMD3100	CXCR4 antagonist	Transwell	5 *μ*g/ml	30 min	Sigma-Aldrich, USA
SP600125	JNK1/2/3 inhibitor	Transwell	10 *μ*M	24 h	Sigma-Aldrich, USA
CXCR4 Ab	Protein analysis	Western blot	2 *μ*g/ml	24 h	Abcam, Cambridge, UK
p-ERK Ab		Western blot	1 *μ*g/ml	24 h	Abcam, Cambridge, UK
p-P38 Ab		Western blot	2 *μ*g/ml	24 h	Abcam, Cambridge, UK
p-JNK Ab		Western blot	1.594 *μ*g/ml	24 h	Abcam, Cambridge, UK
Arpin Ab		Western blot	1 *μ*g/ml	24 h	Abcam, Cambridge, UK
Arp2/3 Ab		Western blot	0.1 *μ*g/ml	24 h	CST, USA

## Data Availability

The data used to support the findings of this study are included within the article.
